# Lipidomic Abnormalities During the Pathogenesis of Type 1 Diabetes: a Quantitative Review

**DOI:** 10.1007/s11892-020-01326-8

**Published:** 2020-08-15

**Authors:** Tommi Suvitaival

**Affiliations:** Steno Diabetes Center Copenhagen, Niels Steensens Vej 2-4, DK-2820 Gentofte, Denmark

**Keywords:** Biomarkers, Lipidomics, Mass spectrometry, Metabolomics, Type 1 diabetes

## Abstract

**Purpose of Review:**

The underlying factors triggering a cascade of autoimmune response that leads to the death of pancreatic beta cells and type 1 diabetes are to large extent unknown. Aberrations in the lipid balance have been suggested, either as factors directly contributing to autoimmunity or as a reflection of external factors, such as the diet or chemical exposure, which may increase the risk or even trigger the autoimmunity cascade.

**Recent Findings:**

A small number of recent studies have investigated the blood lipidome before and after the onset of type 1 diabetes with a goal of identifying biomarkers of disease progression. Phosphatidylcholine levels in particular have been suggested to be reduced prior to the onset of type 1 diabetes.

**Summary:**

In this review, we approach this question through a quantitative analysis of the reported lipids. We quantify the extent of consensus between these heterogeneous studies, describe the overall lipidomic pattern that has been reported, and call for more independent replication of the findings that we highlight in this review.

**Electronic supplementary material:**

The online version of this article (10.1007/s11892-020-01326-8) contains supplementary material, which is available to authorized users.

## Introduction

Type 1 diabetes (T1D) is a chronic disease that starts with an abnormal autoimmune activity that leads to the death of insulin-producing beta cells in the pancreas [1]. Several factors, including viral infections, exposure to toxins, and diet, have been suggested to play role in triggering the autoimmune cascade in some situations or at least in modifying the risk thereof. Also lipids and other circulating compounds have been hypothesized to play a role in the autoimmune cascade, either as a reflection of exposure to external factors such as diet or more directly by creating a persistent state of inflammation in the body due to aberrant lipid homeostasis.

Lipids are a diverse set of molecules that typically consist of one or more fatty acid chains attached to an active head group (see, e.g., Han [2]). For instance, triacylglycerols (or, triglycerides) have three fatty acid chains attached to a glycerol head. This structure, which is a combination of the hydrophobic fatty acid chains and a hydrophilic head group, gives the lipids an ability to self-organize into complex circulating particles that consist of various lipid species. These particles act in tasks of transport and signaling in the body. On the other hand, lipids stored in adipose tissues around the body reflect the long-term state of energy metabolism, and aberrations therein can be observed in obesity [3, 4] and type 2 diabetes [5, 6].

In T1D, very little is known about the role of lipids and other circulating small molecules. At the time of writing, a handful of studies of the lipidome have been published from cohorts from across five different countries and at varying stages of the disease pathogenesis. The greatest interest has been in identifying prospective risk markers for the onset of T1D. This has been investigated using biobank blood samples collected from individuals who during the study follow-up have been diagnosed with T1D. Comparison of the blood lipid profiles of these participants with matched individuals who remained healthy is revealing novel insights into T1D pathogenesis. The majority of the prospective lipidomics studies of T1D have emerged from studies performed in Finland.

Currently, the lipidome is typically measured with mass spectrometry [2], as is the case with all the studies reviewed here. Current lipidomics methods allow for the annotation of several hundred different lipid species from over ten lipid classes (Supplementary Table 1). In addition, thousands of unknown features can be detected. Many of these features are suggested to be noise, but some of these signals may also be of biological relevance even if the chemical structure of the compound is not known. These nontargeted or global lipidomics approaches generate rich data that can be compared between individuals or groups within a study, but do not provide exact measurements of the absolute concentration of a given lipid, which is a prerequisite for a diagnostic tool. Targeted analytical methods with specific standards and calibrations are required for the actual quantification of the compound concentrations, and with the current tools, this is available for at most tens of compounds at any one time.

In this review, we take a quantitative data-driven approach to summarize and interpret the level of consensus between the small and heterogeneous set of studies that currently represent the evidence of disturbances that occur in the lipidome during the pathogenesis of T1D.

## Methods

### Database of Reported Lipidomic Markers in Pathogenesis of Type 1 Diabetes

To identify the relevant publications, results from a Google Scholar search with the query “‘type 1 diabetes’ lipidomics” were evaluated. Relevant publications, reporting findings from lipidomics profiling in human participants prior to or shortly after the onset of type 1 diabetes (T1D), were identified. For each study, the most relevant study question on the pathogenesis of T1D was identified, and results regarding the study question were collected from the publication to a database. The information that was collected is detailed in Supplementary Methods. Critical information about the studies identified is reported in Table 1.

**Table 1:** Summary of the reviewed studies

Study	Country	Age	Time from diagnosis	Contrast	Limiting sample size (median)	Source of results	Number of associated lipids reported	Independence from other studies (%)
La Torre (2013) *Diabetes*	Sweden	Birth		PT1D/CTR	75	Table 3	6	62
Lamichhane (2018) *Scientific Reports*	Finland	6 m, 18 m		PT1D/CTR	25	Fig. 4a	3	0
Lamichhane (2019) *Biomolecules*	Finland	Birth		PT1D/CTR	30	Fig. 1	7	0
Oresic (2008) *Journal of Experimental Medicine*	Finland	Birth, 0–1 y, 1–2 y, 2–3 y, 3–4 y, 4–5 y, 6–7 y, 7–8 y, 8–9 y,9–10 y		PT1D/CTR	30.5	Fig. 3a	39	82
Overgaard (2018) *Metabolomics*	Denmark	< 17 y	1 m, 6 m	Change in C-peptide	123	Table 3, Table 4	27	100
Pflueger (2011) *Diabetes*	Germany	0–2 y, > = 8 y		AB+/AB-	17.5	Supplementary Table 3	15	50
Sen (2020) *Diabetologia*	Finland	1 y, 2 y, 3 y		PT1D/CTR	10	Fig. 1	44	36
Sorensen (2010) *Clinical Biochemistry*	USA	10–29 y	Recent onset	T1D/CTR	10	Table 4	26	100

AB+, antibody-positive; AB-, antibody-negative; CTR, control; m, month; PT1D, progressor to type 1 diabetes; T1D, type 1 diabetes; y, year

Shown in the table are the reference of the study, country of the study cohort, age of the study cohort, time from diagnosis (if applicable), main contrast (or, hypothesis) inspected in this review, the median limiting sample size in testing this hypothesis, source of results in the publication reporting the study, the number of lipids with a reported association in the study, and the level of independence of the list of authors compared to the other seven studies (%)

The database was loaded into R and all consecutive analyses were done in R.

### Consensus Lipidomic Markers in Pathogenesis of Type 1 Diabetes

Table 2 reports associated lipids identified in this analysis. Where results from multiple time points were reported, the median effect specific to the study and lipid species was computed over the time points. Lipid names were simplified to cross-compatible nomenclature using the “map_lipid_names()” function from the lipidomeR package [7]. Then, a lipids-by-studies table of effect signs was created. Lipid species that were reported in at least two studies were included in the table. The rows and the columns of this table were reordered with hierarchical clustering to ease the interpretation of cross-study effect patterns. Lipids with identical cross-study effect pattern were merged to form one row in the table. Finally, the total number of studies, where each lipid was reported, was calculated, and the value was presented as the last column in the table.

**Table 2:** Aberrated lipids that have been reported in at least two of the reviewed studies

Lipids	Oresic (2008) *Journal of Experimental Medicine*	Sen (2020) *Diabetologia*	Overgaard (2018) *Metabolomics*	La Torre (2013) *Diabetes*	Lamichhane (2019) *Biomolecules*	Sorensen (2010) *Clinical Biochemistry*	Pflueger (2011) *Diabetes*	Total
LPC(18:3)	+					+	+	3
TG(51:3)		–			+			2
PC(30:0)		–				+		2
TG(50:2)			–				+	2
CE(18:2)			–		+			2
TG(52:3), TG(52:4)	–		–					2
TG(50:3)	–	–	–					3
CE(18:1), TG(48:3)		–	–					2
TG(46:1)	–				–	+		3
TG(46:2), TG(48:1)	–				–			2
PC(38:6)	–			–				2
PC(36:4)	–	–		–		+	+	5
PC(36:5)	–					+	+	3
PC(32:2)	–					+		2
PC(34:3)	–						+	2
TG(50:1)	–	–					+	3
Cer(40:1), PC-O/P(38:5), TG(51:1), TG(52:5), TG(54:2), TG(54:3), TG(54:5)	–	–						2

CE, cholesterol ester; Cer, ceramide; LPC, lyso-phosphatidylcholine; PC, phosphatidylcholine; PC-O/P, alkyl-acyl PC; TG, triacylglycerol

Shown in the table are the name of the lipid (left), the studies with a study-specific column and indication of the reported aberration (middle; “+” for a positive association, “-“ for inverse association, and empty for no association), and the total number of studies, where the lipid has been reported (right). Lipids (rows) and studies (columns) are sorted by hierarchical clustering to aid interpretation of the lipidomic patterns across the studies

### Similarity Graph of Lipidomics Studies on Type 1 Diabetes

Jaccard similarity was calculated between the lists of reported lipid species from each pair of studies. The pairwise similarity measure was further decomposed into two components: [1] the part of the intersection of the two lists of lipids, where the reported sign of the effect was identical in the two studies, and [2] the remaining part of the intersection, where the reported sign of the effect was the opposite in the two studies. In other words, first, the entire intersection of the lipids reported in two studies was identified for each pair of two studies and, second, it was calculated, what part of these intersecting lipids were in agreement and disagreement in terms of the direction of the aberration. Additionally, Jaccard similarity between the list of lipids reported in each study and the other seven studies was calculated.

**Fig. 1:**
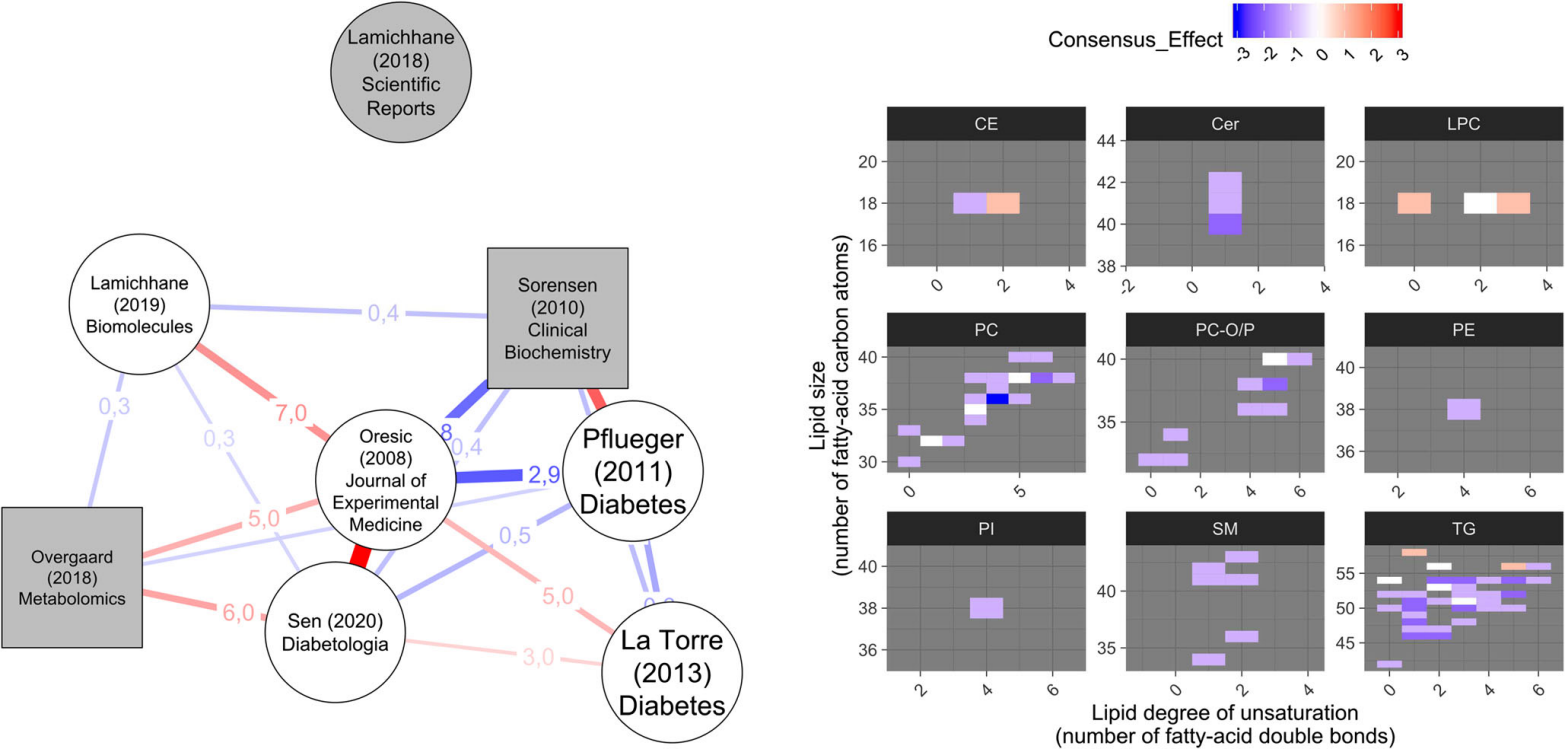
*Left*: Similarity graph of lipidomics studies on T1D onset based on the reported lipidomic associations. Studies are shown as the nodes and they are labeled by the first author, publication year, and the journal. Studies on pre-onset lipidome are shown as round nodes and studies reporting aberrations after the diagnosis of T1D are shown as rectangular nodes. Studies that reported statistical significances after correction for multiple testing are shown as gray nodes, while studies without correction are shown as white nodes. Similarity of two studies is shown the width of the line between them, and their agreement is shown by the color of the line: majority-agreement pairs are shown in red, while majority disagreement pairs are shown in blue. Percentage of agreement and disagreement are shown in the two numbers aside the line (see Methods Section for details). *Right*: Heatmap of consensus aberrations in the lipidome prior to the onset of T1D. Each colored rectangle represents one lipid species, and a deep color indicates an aberration observed in multiple studies (red for positive association between lipid level and progression to T1D; blue for inverse association; gray for not reported). The lipids are categorized into panels by the lipid class (CE, cholesterol ester; Cer, ceramide; LPC, lyso-phosphatidylcholine; PC, phosphatidylcholine; PC-O/P, alkyl-acyl PC; PE, phosphatidylethanolamine; PI, phosphatidylinositol; SM, sphingomyelin; TG, triacylglycerol). Within a lipid-class panel, the individual lipid species are sorted by the lipid size (y-axis) and the degree of unsaturation (x-axis). The phosphatidylcholine PC(36:6) has a highest level of consensus: decreased levels in PC(36:6) have been reported in three studies, as indicated by the deep blue-colored rectangle at position *y* = 36 and *x* = 6 in the PC panel

The similarity graph (Fig. 1, left panel) between the studies was visualized as a network with the qgraph package [8]. In the network, studies are presented as nodes and pairwise similarity between two studies as a line (or, an “edge”) between the two nodes. See Supplementary Methods for details on how the network was constructed. See the caption of Fig. 1 on how to interpret the network.

### Fingerprint of Lipidomic Aberrations Prior to the Onset of Type 1 Diabetes

Finally, studies reporting aberrations before the onset of T1D were specifically analyzed. Pre-onset associations were selected from the database for inspection, and associations relating to post-onset time were omitted. As previously, where results from multiple time points were reported, the median effect specific to the study and lipid species was computed over the time points. Lipid names were simplified to cross-compatible nomenclature using the “map_lipid_names()” function from the lipidomeR package, and lipids were categorized to lipid classes using the same function. Lipid-specific median effects from each study were summed over the studies to create an effect count for each lipid: for instance, a lipid with a reduction in two studies and no change in other studies was summed to a value − 1 + (− 1) = − 2, and a lipid with a reduction in one study, an increase in another study and no change in other studies was summed to − 1 + 1 = 0.

In Fig. 1, right panel, the lipid-specific cross-study effect sums were visualized as a lipidome-wide heatmap using the “heatmap_lipidome()” function in the lipidomeR package in R. The lipidomeR heatmap integrates lipid-specific data as patterns by categorizing lipids by the lipid class and organizing individual lipid species by their size and degree of unsaturation.

## Results

### Database of Reported Lipidomic Markers in Pathogenesis of Type 1 Diabetes

In total, eight publications that report findings from lipidomics profiling in human participants prior to [9••, 10••, 11••, 12••, 13••, 14••] or shortly after [15••, 16••] the onset of type 1 diabetes (T1D) were identified and reviewed. A handful of other publications were identified, reporting associations between the lipidome and external factors such as diet [17] or chemical exposure [18] or disease progression-related aspects, such as the state of the immune system [14••] or development of long-term complications of T1D [19]. However, to our knowledge, the eight studies in this review are the only studies that report direct associations between the lipidome and the disease traits of the T1D pathogenesis. The eight studies are summarized in Table 1.

The majority of these eight studies investigate the time before the onset of T1D. This has been achieved by retrospective analysis of biobank samples from participants who progressed to T1D and participants who remained healthy throughout the follow-up.

For each study, the most relevant study question on the pathogenesis of T1D was identified, and results regarding the study question were collected from the publication. In studies exploring the time before the onset, the most relevant study question was defined as the difference between progressors to T1D and healthy controls. In studies exploring the time after the onset, the most relevant study question was either the decline in insulin secretion [15••] as measured by blood C-peptide or the difference between participants with newly diagnosed T1D and healthy control participants [16••].

Based on the criteria of focus, a total of 449 associations between the blood lipidome and T1D pathogenesis were collected from the publications. The associations covered 114 unique lipid species from 12 lipid classes (Supplementary Table 1). In total, 316 were aberrations prior to the onset of T1D, and 53 were aberrations that relate to the phenotype of the diagnosed disease or to further progression of the disease after diagnosis. The database is available at https://github.com/tommi-s/T1D-lipidome.

### Consensus Lipidomic Markers in Pathogenesis of Type 1 Diabetes

First, the consensus of aberrated lipid species was investigated over all the studies. In total, 26 lipid species were aberrated in at least two of the eight studies. The majority of these lipids were triacylglycerols and phosphatidylcholines with 15 and 6 species, respectively. Other lipid classes with same species reported in at least two studies were two cholesterol esters and one ceramide, lyso-phosphatidylcholine, and alkyl-acyl-phosphatidylcholine.

Next, we describe the reported aberrations in these 26 lipids in more detail. The most frequently reported lipid species was the phosphatidylcholine PC(36:4), which was reported in five of the eight studies. The lipid was reported as reduced prior to the onset of T1D in three studies. In addition, it was reported as elevated in antibody-positive participants compared with antibody-negative participants in another pre-onset study [13••] and positively associated to change in C-peptide in newly-onset T1D [15••].

Other frequently reported lipids were two triacylglycerols, (TG(50:1) and TG(50:3)), the phosphatidylcholine PC(36:5), and the lyso-phosphatidylcholine LPC(18:3). Both the triacylglycerols were reduced in two pre-onset studies. TG(50:1) was also reported elevated in autoantibody-positive participants when compared with autoantibody-negative participants prior to onset of T1D. TG(50:3) was also inversely associated with change in C-peptide after the onset of T1D. PC(36:5) was elevated, both, in autoantibody-positive participants prior to onset of T1D and in newly-onset T1D when compared with healthy controls. In contrast, in a third study, it was reduced in progressors to T1D prior to the onset of the disease. LPC(18:3) was reported in the same three studies as PC(36:5), but it was elevated in all these settings.

The 20 other lipid species were each reported in two studies. The reported associations of the 26 lipid species with pathogenesis of T1D are summarized in Table 2, where the association of each lipid (row) is shown in each study (columns) with a “+” or “-” sign, indicating positive or inverse association, respectively.

### Similarity Graph of Lipidomics Studies on Type 1 Diabetes

After summarizing frequent findings across the studies, we investigated how similar the studies were in terms of the findings. The full similarity graph of the studies is shown in Fig. 1 (left).

The median similarity between two studies was 3%. The highest similarity of 16% was between Oresic et al. [12••] and Sen et al. [14••] (see the widest line in the graph between these two nodes). Both these studies report findings from the DIPP cohort in Finland. Although Pflueger et al. [13••] reported 11% of the same lipid species as Oresic et al., the majority (9%) of these lipids were reported with opposite signs of association (see the second-widest line in the graph between these two nodes, shown in blue to indicate a majority of disagreement between the reported signs of the associations). While seven of the eight studies reported a list of lipids that shared identical species with at least four studies, Lamichhane et al. (2018) [9••] reported a fully unique list of lipids. Although also a DIPP cohort study, none of the lipids reported by Lamichhane, et al. (2018) were confirmed in any of the seven other studies (see the node on top with no lines to the other nodes of the graph).

### Fingerprint of Lipidomic Aberrations Prior to the Onset of Type 1 Diabetes

Finally, we investigated the consensus lipidomic fingerprint prior to the onset of T1D. Differences between progressors to T1D prior to the onset of the disease and healthy controls were reported in four studies. All these studies are based on cohorts in Finland.

In total, 79 lipids were reported in any one of these four studies with a reference to pre-onset. These lipids are from nine lipid classes with the following numbers: 34 species of triacylglycerols (TG), 18 phosphatidylcholines (PC), 11 alkyl-acyl PCs, six sphingomyelins, three ceramides, three lyso-phosphatidylcholines, two ceramides (Cer), two cholesterol esters (CE), one phosphatidylethanolamine, and one phosphatidylinositol.

A minority of these lipids, 17, were reported in two or more of the four studies, while 62 of the 79 lipids were unique to one study. One lipid was reported in three studies: the phosphatidylcholine PC(36:4) was consistently reduced prior to the onset of T1D. Other 16 lipids were reported in two studies: these were Cer(40:1), LPC(18:0), PC(38:6), SM(42:1), and triacylglycerols TG(46:1), TG(46:2), TG(48:1), TG(50:1), TG(50:3), TG(51:1), TG(51:3), TG(52:5), TG(54:2), TG(54:3), and TG(54:5).

Finally, we investigated the aberrated lipidome prior to the onset of T1D by visualizing the reported aberrations on a lipidome-wide heatmap. In the visualization, individual lipid species are categorized into panels by the lipid class and organized on a two-dimensional plane characterizing the size and degree of unsaturation of each lipid species. This approach allows us to interpret association patterns relating to lipid fatty acid chain length and unsaturation.

The 79 lipids reported in the pre-onset studies are shown in Fig. 1 (right panel), where each colored rectangle represents one lipid species and the color of the rectangle represents the consensus of the studies regarding aberration prior to onset of T1D. As indicated by the prevalent blue color, the majority of the aberrations are reductions in the lipid levels prior to the onset of the disease. All reported aberrations in SMs, Cers, PEs, and PIs are reductions in the levels. The majority of aberrations in PCs, PC-O/Ps, and TGs are also reductions. In contrast, only in two LPCs, two TGs and one CE, the consensus aberration is an increase in levels.

Most of the aberrated PCs are polyunsaturated with three or more unsaturated bonds in the fatty acid chains. However, two saturated PCs have been reported. The species reported in more than one studies are polyunsaturated and medium to large sized.

Similarly, TGs primarily are unsaturated with three fully saturated compounds making an exception. All the TGs reported in more than one study are unsaturated medium-sized compounds. The two TGs with increased levels reported in one study are large—in fact, two of the four largest TGs that have been reported prior to the onset of T1D.

In the sphingomyelin pathway, all reported SMs and ceramides are unsaturated and reduced in the level. More specifically, all three ceramides are monounsaturated, and the SMs are equally distributed between monounsaturated and bi-unsaturated compounds.

Both the reported PE and PI are compounds with fatty acid chains consisting of 38 carbons and four unsaturated bonds. Finally, the reported CE and the three reported LPCs all are compounds with 18 carbons in the fatty acid chains.

## Discussion

Results from a total of eight relevant studies were integrated for meta-analysis in this review. A total of 26 lipid species were aberrated during the pathogenesis of type 1 diabetes (T1D) in at least two studies.

Most of the attention on lipids in T1D has focused on phosphatidylcholines and their general inverse association with inflammations of various kinds [20]. However, more than double the number of triacylglycerols as phosphatidylcholines were reported as altered in more than one study. It could be that the triacylglycerols do not reflect the autoimmune response in the same way as the phosphatidylcholines might do. Instead, the triacylglycerols might respond to the decrease in glucose control caused by the beta-cell death, which may already be ongoing although not detectable with clinical relevance.

The hypothesis on relationship between triacylglycerols and the already-decreased glucose control is supported by the observation that many of the reported triacylglycerols are altered after diagnosis, as shown in the two post-onset studies reviewed in this paper. However, not all the reported triacylglycerols are collected from post-onset studies, and even when restricting the analysis to the pre-onset studies, the largest group of altered lipids are the triacylglycerols, as shown in Fig. 1 (right). The chances to inspect this hypothesis further are limited, because neither the glucose levels observed at the lipidomics sampling visits nor comparisons adjusted for the glucose level are available in these publications.

As discussed, the independence of the lipidomic associations from measurable clinical indicators of disease progression is not verified based on the current evidence. The main confounding factor that could explain the associations is the change in glucose control that may occur prior to clinical diagnosis. This is a question that could be addressed in future studies, but it will need larger sample sizes. Considering the great challenges associated with collecting prospective cohorts of relevant size, it is very understandable that the independence has not yet been verified.

Some of the discussed lipids are linked to the immune system in mechanistic studies: fatty acid treatment has been demonstrated to affect the balance of saturated and unsaturated phospholipids in murine macrophages [21]. Autoimmune B cells have been demonstrated to have an altered lipid metabolism, and their self-lipids are aberrated, leading to the hyperactivation of invariant natural killer T (iNKT) cells [22].

Cancer imposes changes in immune system: peroxisome-derived phospholipids, which are elevated in human bone marrow in leukemia, are potential ligands of iNKT cells [23]. It is not known, whether this is linked to the observed reductions in circulating PCs in T1D onset. Furthermore, glycosphingolipids, which are natural ligands for NKT cells, have also been studied: the glucosylceramide synthase inhibitor N-butyldeoxygalactonojirimycin inhibits endogeneous ligands for NKT cells [24]. Isoglobotriaosylceramide (iGb3) is a stimulatory NKT ligand. To our knowledge, these types of glycerosphingolipids have not yet been investigated in T1D onset but would be a particularly interesting avenue for future research.

Changes in SMs could be linked to changes in regulatory T cells (Tregs): acid sphingomyelinase (ASM), which is a rate-limiting enzyme for the breakdown of sphingomyelin into ceramides, is also involved in T cell activity [25, 26]. ASM has been shown to negatively regulate Tregs [27]: first, inhibition of ASM in mice leads to the proliferation of Treg cells and to changes in their phosphorylation. Second, inhibition in the presence of cytokines leads to the proliferation of FOX3P+ induced Tregs. Furthermore, ceramide C6 generally reduces the number of iTregs. The sphingolipid pathway has also been targeted in relation to T cell signaling, providing an opportunity for beneficial immunomodulatory interventions [28]. The bioactive sphingosine-1-phosphate from the sphingolipid pathway has been reported elevated in circulation in two models of T1D [29]. In the same study, the levels of nervonic acid-containing ceramide and sphingomyelin were reduced.

Evidence of alterations of ceramides in population cohorts, which is plentiful in metabolic syndrome and type 2 diabetes (T2D) [30], is still missing in T1D onset. This could partly be explained by the more challenging analysis compared with phospholipids and larger cohorts needed for detection of aberrations, which has been possible earlier with T2D.

In T2D and insulin resistance, ceramides are elevated [30]. It has been debated whether the elevation of fatty acids and ceramides is a cause or a consequence of insulin resistance in the muscle [31]. In animal models, inhibition of de novo ceramide synthesis has demonstrated beneficial effects on lipid metabolism and glucose homeostasis in models of T2D [32, 33]. While changes have been found also in models of T1D [34], the benefit in T1D is yet unclear.

Although only one ceramide and no SMs are reported in more than one of the reviewed T1D onset studies (Table 2), the sphingolipid pathway is affected in later-emerging diabetes complications: SMs are aberrated in diabetic kidney disease [35], and these aberrations modify the risk of mortality in T1D [19]. In the same pathway, ceramides are elevated in diabetic neuropathy but no changes in SMs have been observed [36].

In cohort studies of T1D onset, larger sample sizes are needed for the proper control of the false discovery rate, which has not been done in over half of the studies. In prospective studies, we observed markedly more lipids reported in studies, where false discovery rate was not controlled with correction for multiple testing, when compared with studies, where this was done. Because of the unique new information provided by each of the published studies, however, we did not want to restrict this review only to studies where the false discovery rate was controlled.

The approach in this review was limited to data on effect signs collected manually from result heatmaps or tables. For more accurate cross-study meta-analysis, exactly reported values of the effect sizes and their confidence intervals would be needed, which we call for in future publications. To promote reproducibility and further integration of the existing findings, the database collected for this review is publicly available at https://github.com/tommi-s/T1D-lipidome .

This review on lipidome in the pathogenesis of T1D is limited by the rather small number of studies that have been completed to this date. Considering the broad age interval of the pathogenesis, it is very challenging to design and conduct follow-up studies that investigate the onset of T1D. We thank and congratulate the authors of all the reviewed publications for completing the studies in spite the challenges.

On the other hand, we remark that none of the six reviewed pre-onset studies are completely independent, when evaluating the list of authors. Until the findings are tested in other populations using multiple lipidomics platforms, the lipidomic aberrations preceding the onset of T1D cannot be consider as independently replicated (see Table 1). Regarding aberrations after the onset of the disease, the situation is different but equally challenging: although the studies are independent, there were very few studies that have investigated different questions and, therefore, do not provide opportunity yet for cross-replication.

In this review, our goal was to assess the level of consensus between the studies that have investigated the lipidome in the pathogenesis of T1D and highlight most consistent patterns and individual findings. We propose the phosphatidylcholine PC(36:4) and the 25 other lipids discussed in this review as a starting point for independent replication.

## Conclusion

In total, 449 relevant associations between lipids and pathogenesis of type 1 diabetes (T1D) were reported in eight studies reviewed in this paper. A total of 26 individual lipid species were reported in two or more studies. The majority of these lipids were triacylglycerols and phosphatidylcholines, while two cholesterol esters as well as an individual ceramide and a lyso-phosphatidylcholine also were reported.

Six of the eight reviewed publications provided findings on lipidomic aberrations in the time before the diagnosis of T1D. Here, reductions in the levels of triacylglycerols, phosphatidylcholines, sphingomyelins, and ceramides were reported. Although the cross-study replication of an alteration in individual lipid species was limited to 11 lipids, the pattern in the lipid class in general was in agreement in most of the studies regarding the reduction in phosphatidylcholines and triacylglycerols.

The reduction in the level of the phosphatidylcholine PC(36:4) was identified as the single most consistently replicated finding with a positive result in three out of six studies. We call for more and larger investigations on the nature of these aberrations and propose the lipids highlighted in this review as a starting point for independent replication.

## Electronic Supplementary Material

ESM 1 (DOCX 15 kb)
