# Supplementary material for: Lipidomic Abnormalities During the Pathogenesis of Type 1 Diabetes: a Quantitative Review
Source: Curr Diab Rep. 2020 Aug 15;20(9):46. doi: 10.1007/s11892-020-01326-8 (PMC7429527; doi:10.1007/s11892-020-01326-8)
Supplement: Supplementary file 1 — (DOCX 15 kb) [file 11892_2020_1326_MOESM1_ESM.docx]

## Supplementary Material to

# Lipidomic Abnormalities During the Pathogenesis of Type 1 Diabetes:

# A Quantitative Review

Tommi Suvitaival

[tommi.raimo.leo.suvitaival@regionh.dk](mailto:tommi.raimo.leo.suvitaival@regionh.dk)

Steno Diabetes Center Copenhagen

# Supplementary Tables

Supplementary Table 1: Overview of lipid classes assessed in the review. In the species name, the first number indicates the length of the fatty acid chain(s). The second number indicates the degree of unsaturation (i.e., the number of unsaturated bonds in the fatty acid chain(s).

| **Class abbreviation** | **Class name** | **Number of unique species in the review** | **Example species** |
| --- | --- | --- | --- |
| CE | Cholesterol-ester | 8 | CE(18:1) |
| Cer | Ceramide | 4 | Cer(40:1) |
| DG | Diacylglycerol | 8 | DG(32:1) |
| LPC | Lyso-phosphatidylcholine | 8 | LPC(18:3) |
| LPE | Lyso-phosphatidylethanolamine | 2 | LPE(16:0) |
| PC | Phosphatidylcholine | 21 | PC(36:4) |
| PC-O/P | Alkyl-acyl PC | 12 | PC-O/P(38:5) |
| PE | Phosphatidylethanolamine | 2 | PE(36:2) |
| PE-O/P | Alkyl-acyl PE | 1 | PE-O/P(34:4) |
| PI | Phosphatidylinositol | 1 | PI(38:4) |
| SM | Sphingomyelin | 7 | SM(42:1) |
| TG | Triacylglycerol | 40 | TG(50:1) |

# Supplementary Methods

## Database of Reported Lipidomic Markers in Pathogenesis of Type 1 Diabetes

The following information was collected: name of the reported lipid, description of the comparison (i.e., type of the contrast or group variable used in the statistical comparison), age of the participants at sample collection, time from diagnosis (applicable for post-onset studies), sign of the inferred aberration (i.e., increase or reduction in the level of the lipid), level of statistical significance and whether the test was corrected for multiple testing, country of the study population, the sample size of the case group, the sample size of the control group (if applicable), name of the first author, publication year, publishing journal, source of the information in the publication (i.e., name of the figure or table), URL of the publication, and a full list of the authors.

## Similarity Graph of Lipidomics Studies on Type 1 Diabetes

In the network, studies are presented as nodes and pairwise similarity between two studies as a line (or, an “edge”) between the two nodes. Extent of the similarity is shown by the width of the line. The parts of the intersection decomposed into agreement and disagreement on the sign of the effect are shown by the two values printed over the line: the first value is the percentage of agreement (i.e., where the sign of the effect is identical) and the second value is the percentage of disagreement (i.e., where the sign of the effect is the opposite). Pairs, where the agreement was greater than disagreement, were colored red, and pairs, where the opposite was true, were colored blue. Studies (nodes) were further annotated as follows: First, studies investigating aberrations before the onset of type 1 diabetes were shown as circular nodes and studies investigating aberrations after the onset were shown as rectangular nodes. Second, studies, where associations after correction for multiple testing were reported, were shown with a gray background in the node, and studies, where correction for multiple testing was not reported, were shown with a white background in the node. Finally, the network was laid out using the spring force model, where dissimilar nodes are shown further away from each other whereas similar nodes are pulled closer-by to indicate a tighter relationship.
